# Evaluating newly approved drugs in combination regimens for multidrug-resistant tuberculosis with fluoroquinolone resistance (endTB-Q): study protocol for a multi-country randomized controlled trial

**DOI:** 10.1186/s13063-023-07701-6

**Published:** 2023-11-30

**Authors:** S. B. Patil, M. Tamirat, K. Khazhidinov, E. Ardizzoni, M. Atger, A. Austin, E. Baudin, M. Bekhit, S. Bektasov, E. Berikova, M. Bonnet, R. Caboclo, M. Chaudhry, V. Chavan, S. Cloez, J. Coit, S. Coutisson, Z. Dakenova, B. C. De Jong, C. Delifer, S. Demaisons, J. M. Do, D. Dos Santos Tozzi, V. Ducher, G. Ferlazzo, M. Gouillou, U. Khan, M. Kunda, N. Lachenal, A. N. LaHood, L. Lecca, M. Mazmanian, H. McIlleron, M. Moreau, M. Moschioni, P. Nahid, E. Osso, L. Oyewusi, S. Panda, A. Pâquet, P. Thuong Huu, L. Pichon, M. L. Rich, P. Rupasinghe, N. Salahuddin, E. Sanchez Garavito, K. J. Seung, G. E. Velásquez, M. Vallet, F. Varaine, F. J. Yuya-Septoh, C. D. Mitnick, L. Guglielmetti

**Affiliations:** 1 Indian Council of Medical Research (ICMR) – National AIDS Research Institute, Pune, India; 2 Partners In Health, Maseru, Lesotho; 3 Partners In Health, Astana, Kazakhstan; 4 Institute of Tropical Medicine (ITM), Antwerp, Belgium; 5 Medical Department, Médecins Sans Frontières, 14-34 Avenue Jean Jaurès, 75019 Paris, France; 6 UCSF Center for Tuberculosis, University of California, , San Francisco, San Francisco, CA USA; 7 Epicentre, Paris, France; 8 National TB Center, Almaty, Kazakhstan; 9 National Scientific Center of Phthisiopulmonology, Almaty, Kazakhstan; 10 Université de Montpellier, IRD, INSERM, Montpellier, TransVIHMI France; 11 Médecins Sans Frontières, Mumbai, India; 12 Department of Global Health and Social Medicine, Harvard Medical School, Boston, MA USA; 13 Médecins Sans Frontières, Geneva, Switzerland; 14 City Center of Phthisiopulmonology, Astana, Kazakhstan; 15 Incyte, Morges, Switzerland; 16 Interactive Research and Development (IRD) Global, Singapore, Singapore; 17 Socios En Salud-Sucursal Peru, Lima, Peru; 18 Assistance Publique Hôpitaux de Paris (APHP), Unité de Recherche Clinique, Hôpital Pitié-Salpêtrière, Paris, France; 19 Santé Arménie French-Armenian Research Center, Yerevan, Armenia; 20 Division of Clinical Pharmacology, Department of Medicine, University of Cape Town, Cape Town, South Africa; 21 Wellcome Centre for Infectious Diseases Research in Africa (CIDRI-Africa), Institute of Infectious Disease and Molecular Medicine, University of Cape Town, Cape Town, South Africa; 22 Medicines Patent Pool, Geneva, Switzerland; 23 Indian Council of Medical Research Headquarters, New Delhi, India; 24 Indian Journal of Medical Research, New Delhi, India; 25 Hanoi Lung Hospital, Hanoi, Vietnam; 26 Partners In Health, Boston, MA USA; 27 Division of Global Health Equity, Brigham and Women’s Hospital, Boston, MA USA; 28 Indus Hospital & Health Network, Karachi, Pakistan; 29 Hospital Nacional Sergio E. Bernales, Lima, Peru; 30 Division of HIV, Infectious Diseases, and Global Medicine, University of California, San Francisco, San Francisco, CA USA; 31 Brigham and Women’s Hospital, Boston, MA USA; 32 Sorbonne Université, INSERM, U1135, Centre d’Immunologie Et Des Maladies Infectieuses, Paris, France; 33 Assistance Publique Hôpitaux de Paris (APHP), Groupe Hospitalier Universitaire Sorbonne Université, Hôpital Pitié Salpêtrière, Centre National De Référence Des Mycobactéries Et De La Résistance Des Mycobactéries Aux Antituberculeux, Paris, France

**Keywords:** Fluroquinolone-resistant, Pre-XDR TB, Multidrug-resistant, Rifampicin-resistant, MDR-TB, RR-TB, Tuberculosis, Bedaquiline, Clofazimine, Delamanid, Linezolid, Treatment shortening, Non-inferiority, Stratified medicine

## Abstract

**Background:**

Treatment for fluoroquinolone-resistant multidrug-resistant/rifampicin-resistant tuberculosis (pre-XDR TB) often lasts longer than treatment for less resistant strains, yields worse efficacy results, and causes substantial toxicity. The newer anti-tuberculosis drugs, bedaquiline and delamanid, and repurposed drugs clofazimine and linezolid, show great promise for combination in shorter, less-toxic, and effective regimens. To date, there has been no randomized, internally and concurrently controlled trial of a shorter, all-oral regimen comprising these newer and repurposed drugs sufficiently powered to produce results for pre-XDR TB patients.

**Methods:**

endTB-Q is a phase III, multi-country, randomized, controlled, parallel, open-label clinical trial evaluating the efficacy and safety of a treatment strategy for patients with pre-XDR TB. Study participants are randomized 2:1 to experimental or control arms, respectively. The experimental arm contains bedaquiline, linezolid, clofazimine, and delamanid. The control comprises the contemporaneous WHO standard of care for pre-XDR TB. Experimental arm duration is determined by a composite of smear microscopy and chest radiographic imaging at baseline and re-evaluated at 6 months using sputum culture results: participants with less extensive disease receive 6 months and participants with more extensive disease receive 9 months of treatment. Randomization is stratified by country and by participant extent-of-TB-disease phenotype defined according to screening/baseline characteristics. Study participation lasts up to 104 weeks post randomization. The primary objective is to assess whether the efficacy of experimental regimens at 73 weeks is non-inferior to that of the control. A sample size of 324 participants across 2 arms affords at least 80% power to show the non-inferiority, with a one-sided alpha of 0.025 and a non-inferiority margin of 12%, against the control in both modified intention-to-treat and per-protocol populations.

**Discussion:**

This internally controlled study of shortened treatment for pre-XDR TB will provide urgently needed data and evidence for clinical and policy decision-making around the treatment of pre-XDR TB with a four-drug, all-oral, shortened regimen.

**Trial registration:**

ClinicalTrials.Gov NCT03896685. Registered on 1 April 2018; the record was last updated for study protocol version 4.3 on 17 March 2023.

**Supplementary Information:**

The online version contains supplementary material available at 10.1186/s13063-023-07701-6.

## Background and rationale

The endTB-Q trial (Evaluating Newly approved Drugs in combination regimens for multidrug-resistant Tuberculosis with fluoroquinolone resistance, ClinicalTrials.Gov identifier NCT03896685) was designed in 2019 to test recently developed drugs in combination with repurposed drugs for multidrug-resistant/rifampicin-resistant tuberculosis (MDR/RR-TB). According to the World Health Organization (WHO) Global TB Report, there were an estimated 484,000 (range, 417,000–556,000) new cases of multidrug-resistant/rifampicin-resistant tuberculosis (MDR/RR-TB) and 214,000 (range, 133,000–295,000) total deaths from this disease in 2018 [1]. Among MDR/RR-TB patients tested for resistance to fluoroquinolones, 20.8% (95% confidence interval [CI] 16.3–25.8%) had resistance to any fluoroquinolone (pre-extensively drug-resistant TB, pre-XDR TB) [2]. A 2016 publication of a multi-country survey found resistance to ofloxacin in the same range, 12.3 to 30.7% of RR-TB patients, though prevalence varied by country [3]. At the time, the efficacy of the standard-of-care treatment for MDR/RR-TB was unsatisfactory, with favorable treatment outcomes achieved in 56% of patients according to the WHO Global TB Report and in 54–62% of patients in previous studies [1, 4–6]. Treatment outcomes were noticeably worse in the presence of resistance to any fluoroquinolone [7, 8]. In a multi-country study including 1433 patients, only 33% of patients harboring fluoroquinolone-resistant MDR-TB strains achieved favorable treatment outcomes compared to 60% in MDR-TB patients with no resistance to any second-line drug [9].

Treatment for pre-XDR TB continued to be longer than treatment for fluoroquinolone-susceptible MDR/RR-TB. While a shorter, 9–12-month regimen was recommended by WHO for the latter group as early as 2016, patients with pre-XDR TB were not eligible for this regimen [10, 11]. This group saw significantly improved outcomes with increasing use of new (bedaquiline, delamanid) and repurposed (clofazimine, linezolid) drugs as part of conventional regimens [12–18]. These longer regimens often relied on intramuscular or intravenous agents including second-line injectables and carbapenems and were burdened by substantial drug-associated toxicity. This included ototoxicity, liver and renal toxicity, gastrointestinal disorders, electrolyte imbalance, hypothyroidism, and neurotoxicity. The occurrence of these adverse events led to frequent suspension and replacement of the suspected drug(s) in conventional regimens [19, 20]. In addition, these adverse events have been shown to be one of the major drivers of treatment default [20, 21], as well as to be associated with a lower rate of culture conversion [22]. Monitoring and management of adverse events over long treatment courses engenders substantial costs for patients and health systems [10, 23]. Linezolid, in particular, has been identified as one of the drugs which is most frequently associated to adverse events in MDR/RR-TB regimens: optimizing its posology is a key research priority [24].

When launched in 2020, endTB-Q was the first randomized, internally controlled trial to evaluate a short, all-oral regimen exclusively among patients with pre-XDR TB, with the goal of improving the level of evidence supporting treatment for this neglected population.

## Objectives

The primary objective of the endTB-Q trial is to assess whether the efficacy of the experimental arm at week 73 is non-inferior to that of the control. Secondary objectives are as follows: (1) to compare the efficacy of the experimental arm at week 104 to that of the control; (2) to compare the frequency of and time to early treatment response (culture conversion) in the experimental arm to that of the control; (3) to compare the efficacy of the experimental arm at week 39 to that of the control; (4) to compare, at week 73 and week 104, the proportion of participants who experience failure or relapse in the experimental arm to that in the control arm; (5) to compare, at week 73 and week 104, the proportion of participants who die of any cause in the experimental arm to that in the control arm; (6) to compare, at week 73 and week 104, the proportion of participants who experience grade 3 or higher adverse events (AEs) or serious AEs (SAEs) of any grade in the experimental arm to that in the control arm; and (7) to describe, at week 73 and week 74, the proportion of participants who experience adverse events of special interest (AESI) in the experimental arm and in the control arm. Here we describe version 4.3 of the endTB-Q protocol. The safety and efficacy of two linezolid dose-reduction strategies are the subject of exploratory objectives.

## Methods & analysis

The study protocol hereby presented contains all items defined by the Standard Protocol Items: Recommendations for Interventional Trials (SPIRIT) statement. A completed SPIRIT checklist is provided (Supplement 1).

### Trial design

**Fig. 1:**
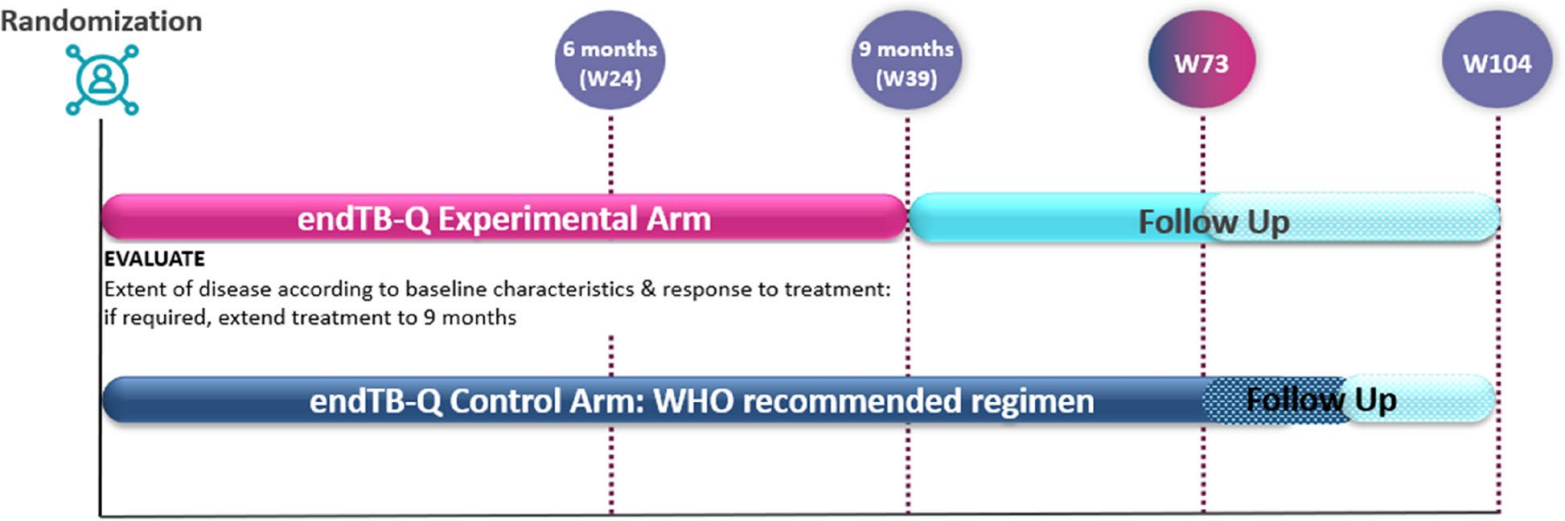
Study schema

endTB-Q is a randomized, controlled, open-label, multi-country Phase III trial evaluating the efficacy of a new combination regimen and strategy for the treatment of pre-XDR TB. The study will enroll a total of 324 participants in parallel across one experimental and one standard-of-care control arms, in a 2:1 ratio. Study participation will last up to 104 weeks post randomization; however, those participants remaining in follow-up will have their follow-up terminated when the last participant completes 73 weeks (“hybrid” follow-up duration) (Fig. 1).

### Study setting

The endTB-Q trial is sponsored by Médecins Sans Frontières (MSF) France and jointly coordinated by members of the endTB consortium, Interactive Research and Development (IRD), MSF, and Partners In Health (PIH), and their research partners, Harvard Medical School, Epicentre, the Institute of Tropical Medicine of Antwerp (ITM), and the University of California San Francisco (UCSF). The trial is implemented in countries selected for the following: a significant burden of MDR/RR-TB with resistance to fluoroquinolones; the presence of a member institution of the endTB consortium or another entity experienced in TB clinical trials, and an existing relationship between TB services and the endTB consortium and partners; clinical trial experience or potential (established through a multi-step site assessment process); suitable MDR-TB clinical management systems, regulatory environment, research pharmacy capability, and microbiology/molecular biology services; and heterogeneity in DR-TB patient characteristics (geography, resistance, comorbidities, risk-factor profiles). endTB-Q participants are recruited in India, Kazakhstan, Lesotho, Pakistan, Peru, and Vietnam.

### Study population

#### Eligibility criteria

##### Inclusion/exclusion

Adults and adolescents (≥ 15 years old) with pulmonary TB with intolerance to fluoroquinolones or with disease caused by *Mycobacterium tuberculosis* resistant to rifampicin and not susceptible to fluoroquinolones. Table 1 summarizes trial inclusion and exclusion criteria.

**Table 1:** Study inclusion and exclusion criteria

Inclusion criteria	Exclusion criteria
• Documented pulmonary TB due to strains of *Mycobacterium tuberculosis* resistant to rifampicin and resistant to fluoroquinolones, by a validated rapid molecular test. In trial sites in countries with high background prevalence of fluoroquinolone resistance (India and Pakistan), an undefined^a^ result of a validated rapid molecular tests for fluoroquinolone resistance also permits inclusion. Patients with RR-TB who are unable to tolerate fluoroquinolones are also eligible, regardless of resistance or susceptibility to fluoroquinolones • ≥ 15 years of age • Willingness to use contraception • Provision of informed consent for study participation • Residence in a dwelling that can be located by study staff and an expectation to remain in the area for the duration of the study	• Patients with known allergies or hypersensitivity to any of the investigational drugs • Patients known to be pregnant or unwilling or unable to stop breastfeeding an infant • Patients unable to comply with treatment or follow-up schedule • Patients with exposure (intake for 30 days or more) in the past 5 years to bedaquiline, delamanid, linezolid, or clofazimine, or with proven or likely resistance to bedaquiline, delamanid, linezolid, or clofazimine • Patients who have received second-line drugs for 15 days or more prior to the screening visit date in the current MDR-TB treatment episode; exceptions include (a) patients who have experienced treatment failure, (b) patients who are restarting treatment after having been lost to follow-up, and (c) treatment adaptation to adapt to new WHO treatment recommendations • Patients with one or more of the following laboratory results: o Grade 3 or higher hemoglobin, calcium, magnesium, creatinine, or bilirubin o Grade 2 or higher potassium, aspartate aminotransferase, alanine aminotransferase, or total bilirubin o Grade 4 result on any other screening laboratory tests • Patients with cardiac risk factors including ECG abnormalities (i.e. QTcF ≥ 450 ms), pacemaker implant, and personal history of cardiovascular disease (i.e. long QT syndrome, left or right bundle branch block) or family history of long QT syndrome • Patients requiring continued use of a contraindicated medication • Patients currently taking part in another trial of any medication used or being studied for TB treatment • Patients with any condition (social or medical) which, in the opinion of the investigator, would make study participation unsafe

^a^Undefined combines *Mycobacterium tuberculosis* not detected and indeterminate for fluoroquinolone wild-type or mutant

##### Randomization

Patients are randomized 2:1 (experimental to control) at inclusion in the study. Randomization is stratified by country and by participant extent-of-TB-disease phenotype defined according to screening/baseline characteristics, as detailed below. Randomization is blocked, using blocks of varying size. Allocation concealment is ensured by random sequence generation. Once a participant is eligible for the study and his/her details are entered into the clinical trial database, the Site Investigator responsible for randomization receives the participant inclusion number and the allocated regimen through a centralized interactive randomization system. Being an open-label trial, the regimen is not masked from participants and Site Investigators. However, microbiology staff who perform testing and Central Investigators are blinded to treatment assignment. Since Site Investigators are not blinded to assignment and are possibly influenced by opinions about regimen allocation, permanent regimen changes are made with input from the independent Clinical Advisory Committee (CAC), staffed by expert MDR-TB clinicians. The CAC also validates study outcomes that are assigned by Site Investigators. CAC members do not provide any input on the study protocol and are not involved in the study analysis. A secondary, balanced (1:1 allocation ratio) randomization to a linezolid dose-reduction strategy (300 mg daily or 600 mg thrice weekly) is performed for participants in the experimental arm at 16 weeks post randomization, or earlier if required for toxicity.

##### Procedure for unblinding

The design of the study is open label: therefore, unblinding will not occur.

##### Treatment arms and duration

The experimental arm regimen includes bedaquiline, clofazimine, delamanid, and linezolid. Drugs in the experimental arm are dosed according to prespecified weight bands (Table 2). Linezolid dosing starts at 600 mg/day; the aforementioned dose-reduction randomization assigns experimental arm participants to receive either linezolid at 300 mg daily or at 600 mg thrice weekly starting at 16 weeks (or earlier, if indicated by toxicity). Treatment duration in the experimental arm is 24 or 39 weeks, according to the participant extent-of-TB-disease phenotype at screening/baseline and treatment response prior to 24 weeks. Treatment duration will be evaluated at baseline according to extent-of-TB-disease phenotype, classified according to highest grade of sputum smear at screening and presence/absence of any lung cavity on baseline chest radiograph (Table 3). In addition, culture results on sputum specimens collected at week 8 and later will be assessed at the time of the week 24 visit. If any of the following is true, treatment duration will be 39 weeks: (a) treatment duration was assigned to be 39 weeks based on screening smear and baseline chest radiograph; (b) there is ≥ 1 positive culture result from sputum specimens collected at week 8 or all culture results from sputum specimens collected at week 8 are missing or contaminated; or (c) any culture is positive from a sputum specimen collected after week 8, with result available at the time of the week 24 visit. If none of the above is true, participants will receive 24 weeks of treatment. Participants may take as long as 32 weeks to complete all doses of a 24-week treatment regimen, and up to 47 weeks to complete all doses of a 39-week treatment regimen.

**Table 2:** Drug dosing for the endTB-Q experimental arm

Drug	Weight band (kg)	
	**24–30**	** > 30**
**Bedaquiline**	200 mg daily for 2 weeks followed by 100 mg 3 × /week	400 mg daily × 2 weeks followed by 200 mg 3 × /week
**Clofazimine**	100 mg daily	100 mg daily
**Delamanid**	50 mg twice daily	100 mg twice daily
**Linezolid**^**a**^	300 mg daily up to week 16 (followed by 300 mg daily or 600 mg 3 × /week)	600 mg daily up to week 16 (followed by 300 mg daily or 600 mg 3 × /week)

^a^Linezolid dosing is routinely modified at week 16, or sooner if necessary, to reduce toxicity related to linezolid. The modification is either decreased (300 mg daily) or intermittent (600 mg 3 × /week) dosing as defined by a balanced secondary randomization

**Table 3:** Duration of treatment according to extent-of-TB-disease phenotype at screening/baseline

Any lung cavity	Screening sputum AFB smear grade			
	Negative or scanty	1 +	2 +	3 +
Cavity absent	24 weeks of treatment^a^	24 weeks of treatment^a^	39 weeks of treatment	39 weeks of treatment
Cavity present	24 weeks of treatment^a^	39 weeks of treatment	39 weeks of treatment	39 weeks of treatment

Abbreviation: *AFB* acid-fast bacilli. Screening sputum AFB smear grade is assigned based on the highest smear grade from a screening (or baseline) sputum sample (*N* = 3). Cavitary disease is defined as the presence of at least one lung cavity on the baseline chest radiograph

^a^These participants might have treatment extended to 39 weeks based on treatment response, defined by sputum culture results assessed at week 8 or between week 8 and week 24

Treatment regimens in the control arm are constructed according to latest WHO recommendations and local guidance: composition of the regimens may therefore change over the course of the trial [25–27]. Duration is variable: the conventional regimen is delivered for approximately 78 weeks. Oral drugs are delivered 7 days/week in both experimental and control arms. Injectable drugs (rarely used) in the control arm are delivered at least 6 days/week, according to local practices. Drug intakes are directly observed.

##### Study treatment discontinuation and study withdrawal

Study treatment may be discontinued in the following situations: (1) pregnancy or breastfeeding, (2) required use of prohibited concomitant medications, (3) indications of treatment failure, and (4) any other condition (social or medical) which the Site Principal Investigator believes would make study participation unsafe. Study treatment discontinuation is defined as permanent discontinuation of two or more investigational drugs, or addition or replacement of one or more investigational drugs in the experimental arm; and as addition or replacement of two or more drugs in the control arm. Prohibited concomitant medications depend on the treatment received by the participant. They include moderate and strong CYP3A4 inhibitors and inducers for bedaquiline-containing regimens; strong inducers are also disallowed with delamanid-containing regimens. With linezolid-containing regimens, disallowed medications are any medicinal product that inhibits monoamine oxidases A or B, tricyclic antidepressants, selective serotonin reuptake inhibitors, selective serotonin/norepinephrine reuptake inhibitor, triptans, and other serotoninergic agents. Decisions to permanently discontinue study treatment are taken in consultation with the CAC. Participants are referred to local services for treatment and an early termination visit is performed. In addition, participants discontinuing treatment before the week 73 visit perform post-termination follow-up visits at weeks 39 and 73, as needed. Participants who withdraw consent will be withdrawn from the study.

##### Recruitment and retention

Prospective participants are identified by facility staff in inpatient or outpatient TB diagnosis and/or treatment facilities located in the study catchment areas. Patients who agree to be evaluated for the study are referred to study staff. Study staff explain the study, including potential risks and benefits associated with participation. Subsequently, screening consent is obtained from participants (or from parent or guardian, in case of minors, who also provide assent) by Site Investigators or other delegated site staff prior to any trial-specific evaluation. Baseline consent and randomization follow in those who are eligible. Retention in the study is ensured through comprehensive, individualized participant support, including adherence enablers and home visits as needed. During treatment, adherence is monitored at every visit and adherence counselling is provided by specialized staff. All transportation costs for study participation are covered by the study. Food support is provided. Participants requiring care for comorbidities (e.g., HIV, diabetes mellitus) receive care in the study setting or through facilitated referrals to local providers. Care for adverse events is provided through the same channels, at no cost to study participants. The sponsor has insurance to cover for non-negligent harm associated with the protocol. Participants requiring ongoing treatment for TB after trial participation receive care through facilitated referrals to local providers; study drugs are available in these settings. The participant information materials and informed consent form are available from the corresponding author on request.

### Outcomes

#### Efficacy

The primary efficacy outcome is the proportion of participants with favorable outcome at week 73, as defined in Table 4.

**Table 4:** Primary efficacy outcome definitions

Favorable outcome	Unfavorable outcome
If the outcome is not classified as unfavorable, and one of the following is true: 1. The last two culture results are negative. These two cultures must be taken from sputum samples collected on separate visits, the latest between weeks 65 and 73 2. The last culture result (from a sputum sample collected between weeks 65 and 73) is negative, and either there is no other post-baseline culture result or the penultimate culture result is positive due to laboratory cross contamination, and bacteriological, radiological and clinical evolution is favorable	If any of the following occur: 1. Replacement or addition of one or more investigational drugs in an experimental arm (failure) 2. Replacement or addition of two or more investigational drugs in the control arm (failure) 3. Initiation of a new MDR-TB treatment regimen after the end of the allocated study regimen and before week 73 (recurrence) 4. Death from any cause 5. At least one of the last two cultures, the latest being from a sputum sample collected between weeks 65 and 73, is positive in the absence of evidence of laboratory cross contamination (failure/recurrence) 6. The last culture result (from a sputum sample collected between weeks 65 and 73) is negative; AND there is no other post-baseline culture result or the penultimate culture is positive due to laboratory cross contamination; and bacteriological, radiological, or clinical evolution is unfavorable (failure/recurrence) 7. There is no culture result from a sputum sample collected between weeks 65 and 73 or it is positive due to laboratory cross contamination AND the most recent culture is negative; and bacteriological, radiological, or clinical evolution is unfavorable (failure/recurrence); or the most recent culture result is positive in the absence of laboratory cross contamination 8. The outcome is not assessable because there is no culture result from a sputum sample collected between weeks 65 and 73 or it is positive due to laboratory cross contamination AND - there is no other post-baseline culture result or the most recent culture is positive due to laboratory cross contamination; or - the most recent culture is negative and bacteriological, radiological, and clinical evolution is not assessable 9. Previously classified as unfavorable in the present study (except for participants whose outcome at 39 weeks was unfavorable because it was unassessable)

The secondary efficacy outcomes are the following:

1. The proportion of participants with favorable outcome at week 39;
2. The proportion of participants with favorable outcome at week 104;
3. The proportion of participants who experienced failure or relapse at week 73 and at week 104;
4. Early treatment response, which is assessed through the following:
  - Proportion of participants with culture conversion at 8 weeks assessed in Mycobacteria Growth Indicator Tube (MGIT) culture method (and on Löwenstein-Jensen [LJ] culture medium where possible);
  - Time to culture conversion assessed in MGIT system (and LJ where possible); and
  - Change in time to positivity (TTP) in MGIT over 8 weeks.

Efficacy endpoints at weeks 39, 73, and 104 are validated by the CAC. Although differences are not expected, the primary efficacy endpoints are also used to evaluate efficacy across linezolid dose-reduction strategies.

#### Safety

The secondary safety outcomes are the following:

1. At week 73 and week 104, the proportion of participants who died of any cause;
2. The proportion of participants with grade 3 or greater AEs and serious adverse events (SAEs) of any grade by week 73 and by week 104;
3. The proportion of participants with AESIs by week 73 and by week 104.

The endpoint for assessment of safety of the linezolid dose-reduction strategies is severe linezolid-related toxicity, defined as grade 3 or higher linezolid-related AEs (leukopenia, anemia, thrombocytopenia, peripheral neuropathy, and optic neuropathy), SAEs, and AEs requiring linezolid discontinuation.

### Schedule of events

**Fig. 2:**
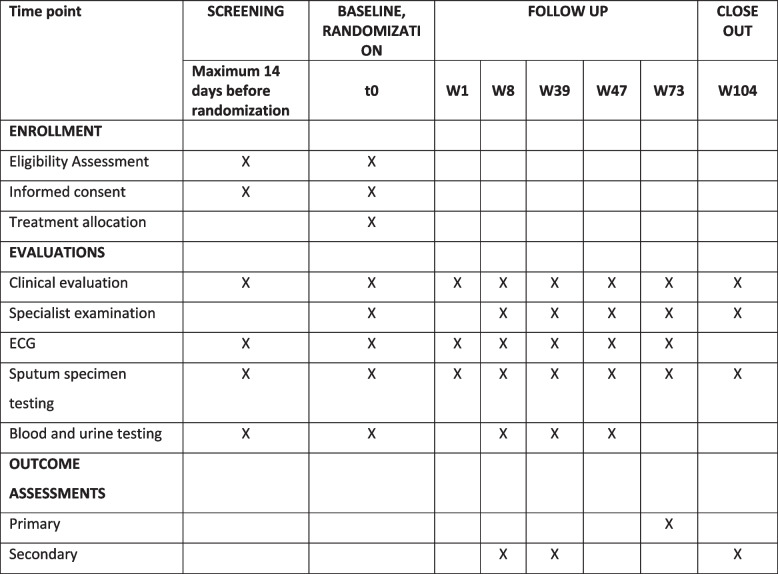
Summary schedule of events and study procedures

Figure 2 outlines the schedule of events and procedures undertaken during study participation.

### Adverse events and pregnancy

AEs are assessed by the study clinicians at all study visits. Spontaneous reporting of adverse events can also occur at scheduled study visits, through daily treatment support, or at unscheduled visits. Adverse events are managed according to grade and relatedness to study drug; closer monitoring may be recommended at any grade. Investigators are encouraged to modify or withhold study drugs possibly related to adverse events of grade 3 or higher.

Severity is graded according to the standardized MSF Severity Grading Scale, which was developed using the Division of Microbiology and Infectious Diseases (DMID) adult toxicity tables (November 2007) and the Common Terminology Criteria for Adverse Events v. 4.03 (CTCAE) (June 2010). The following AEs, regardless of their seriousness or causal relationship to treatment, are considered of interest: (a) Grade 3 or above “electrocardiogram QT corrected interval prolonged”; (b) Grade 3 or above leukopenia, anemia, or thrombocytopenia; (c) Grade 3 or above peripheral neuropathy; (d) Grade 3 or above optic neuritis; and (e) Grade 3 or above increase in alanine aminotransferase (ALT) or aspartate aminotransferase (AST). AEs are managed according to grade and relatedness to study drugs; closer monitoring may be recommended at any grade. Investigators are encouraged to modify or withhold study drugs possibly related to AEs of grade 3 or higher. Additional guidance is provided in study standard operating procedures and by the CAC.

If a study participant (or their partner) is found to be pregnant while being treated with the investigational drugs or during the safety follow-up period, pregnancy is notified to the pharmacovigilance (PV) unit and followed up until a pregnancy outcome is known. Infants born from exposed pregnancies are followed up at least at 6 and 12 months of age and assessed for fetal/child anomaly, birth defect, or other serious consequence. People who become pregnant during study participation and whose pregnancy is not terminated may remain on study treatment if all the following conditions are met: (a) the clinical trial insurance policy in the country covers participant pregnancy, including damage to and loss of the fetus; (b) the local authorities and ethics committee(s) approve; (c) the participant is at least 18 years of age; (d) for the individual participant, the Site Principal Investigator, according to his/her clinical judgment, considers that the expected benefits of continuing the treatment outweigh the risks of ongoing fetal exposure; (e) the CAC agrees with the Site Principal Investigator’s recommendation; and (f) the participant is informed of the therapeutic options and her separate specific consent is obtained.

### Data collection, monitoring, and management

Data are collected and entered into an electronic data capture system (OpenClinica v.3.16, OpenClinica LLC. Waltham, MA, USA) in a web-based system that is compliant with International Conference on Harmonisation (ICH) Good Clinical Practice (GCP) guidelines. Each participant is assigned a unique study identifier. Designated study team members at each participating site perform real-time quality control and periodic quality assurance activities. Checks for consistency are implemented at the data entry level on site and centrally after data entry. Regular data review and data cleaning for quality control are organized in a blinded way. In addition, external monitoring is performed in accordance with the protocol specific requirements, ICH GCP guidance, and other applicable requirements.

Data are managed centrally by Epicentre. Additionally, safety data are also entered in a separate PV database at the centralized MSF PV Unit. Appropriate medical and research records are maintained for the trial, in compliance with ICH GCP and regulatory and institutional requirements. All study documents are coded with a study identification number. All study records are managed in a secure and confidential fashion. All AEs that occur during study are documented and followed to resolution or stabilization; in the case of AESIs and SAEs, this follow-up may extend beyond the normal study reporting period. SAEs are notified, within 24 h of awareness, by the Site Principal Investigator (or designee) to the MSF PV Unit. All SAEs deemed related to one or more investigational product(s) and considered unexpected with the use of such products are reported to National Regulatory Authorities and national/local IRBs. All other SAEs are reported in an Annual Safety Report prepared by the MSF PV Unit, and earlier if there are specific local regulations for more frequent reporting.

Safety oversight is under the direction of the independent Data Safety Monitoring Board (DSMB), the members of which have expertise in clinical trials, MDR-TB, pharmacology, and electrophysiology. The DSMB reviews safety and efficacy data on each arm of the study at least semi-annually and provides recommendations to the study Sponsor. The DSMB also receives listings and/or reports of SAEs, AESIs, and pregnancies notified since the last DSMB meeting. The DSMB may recommend study termination in case of unacceptable toxicities or unequivocal efficacy results.

### Sample size

The sample size calculation required assumptions about the primary outcome frequency at week 73 for the experimental and control arms, the type I error, and the non-inferiority margin. Estimates of response of fluoroquinolone-resistant MDR-TB to regimens containing bedaquiline and/or delamanid came from available published observational cohorts [10, 11, 28–30] for conventional 18–24-month treatment regimens, and from NiX-TB for shorter regimens [31]. Overall, five observational cohorts included a pooled sample of 861 patients, with 618 (72% [95% CI 69–75%]) who achieved a favorable outcome [9, 10, 28–30]. At the time of the sample size calculation, Nix-TB reported 88% (95% CI 78–94%) success (among 75 participants eligible for 6 months of post-treatment follow-up) with a regimen that could be considered similar to the endTB-Q experimental regimen. We therefore assumed a 73-week treatment response of 78% (the lower bound of the 95% CI around the point estimate of treatment success in NiX-TB) in the experimental arm and 75% (corresponding to the upper bound of the 95% CI around the point estimate of treatment success for the longer conventional regimens containing newly approved drugs) in the control arm. This calculation was conservative, in that it assumed a relatively small difference in treatment response. With a 12% non-inferiority margin and alpha set to 2.5% (one-sided), assumed loss of 6% of subjects between the randomized population and modified intent-to-treat (mITT) population and an additional loss of 10% between the mITT and per-protocol (PP) populations, and a 2:1 allocation ratio between experimental and control arm, a sample size of 324 randomized participants provides power greater than 80% to show the non-inferiority in both the mITT and PP populations. The sample size calculations were performed using Power Analysis and Sample Size Software (v.4, NCSS, LLC. Kaysville, UT, USA).

#### Rationale for the non-inferiority design

The current study aims to make the following improvements over the conventional regimen for pre-XDR TB: (a) shorten treatment from 18–24 months to 6–9 months; (b) eliminate the injectable and establish an all-oral regimen; (c) reduce the toxicity profile, including for patients coinfected with HIV; and (d) enhance treatment adherence and completion. Achieving these four objectives, even without improving the efficacy of the current regimen, would confer benefits to populations as well as to individual patients. It may also improve compliance with treatment. Improved compliance, in turn, could translate into reduced frequency of loss-to-follow-up of patients on treatment. Both these changes would have important epidemiological implications, reducing transmission of and morbidity and mortality from MDR-TB. In addition, shorter, less-toxic regimens could engender quality-of-life and economic benefits for patients who are able to return to activities of daily life sooner. A modest loss in efficacy could be accepted in exchange for easier delivery, shorter duration, and improved tolerability. In light of these potential considerable benefits, even without an improvement in efficacy, a non-inferiority design was selected for the endTB-Q trial.

#### Rationale for the choice of the non-inferiority margin

We elected a 12% non-inferiority margin for several reasons. First, the comparator in endTB-Q reflected an important improvement over the commonly used standard of care in 2019 by including at least one new drug. Consequently, the expected proportion of favorable outcomes in the control arm reflected an increase of more than 40% over the standard reported in the WHO Global TB Report. Concern about bio-creep is, therefore, mitigated. Second, relative to the control arm (and the WHO-recommended longer regimen), the experimental regimen would result in a significantly reduced pill burden and treatment duration, expected better tolerability, and expected increased adherence achieved by reducing the treatment duration from more than 100 weeks (in the control) to 39 or 24 weeks (in the experimental arm). Lastly, the 12% margin has been used in two other novel-regimen studies: (1) STAND, which was vetted by both the US FDA and the EMA [29]; and (2) endTB clinical trial, which has been approved by the MSF ERB, as well as IRBs in 8 countries, including the USA and Belgium [30]. STAND was primarily a study of a new regimen for drug-susceptible TB. Arguably, since current treatment for drug-susceptible TB is very effective and well tolerated, there would be little tolerance for a new regimen with any decrease in efficacy. In contrast, the control in endTB-Q is at least 18 months and may be poorly tolerated. A modest loss in efficacy could be accepted in exchange for easier delivery, shorter duration, and improved tolerability.

### Analysis of the primary endpoint and analysis populations

#### Analysis populations

The safety population will include all enrolled participants who receive at least one dose of study treatment (exposed). Safety analyses will be based on the treatment actually received after inclusion (as treated). The first efficacy population will be the mITT population containing all randomized participants with culture-positive, rifampicin-resistant TB in whom fluoroquinolone susceptibility has been ruled out. Participants whose sputum culture is not positive for *M. tuberculosis* will be excluded from the mITT population. Exclusion from the mITT population will occur if screening/baseline DST results from the designated study lab indicate resistance (using a test deemed to be reliable by the trial reference lab, ITM) to a drug contained in the experimental regimen. Participants with an undefined fluoroquinolone resistance test at screening/baseline will be excluded from the mITT population if a subsequent test (phenotypic or genotypic) finds fluoroquinolone susceptibility. Participants without any post-baseline data will be also excluded from the mITT population. Participants in the mITT population will be analyzed in the arm to which they were randomly allocated (as randomized). The PP population is the same as the mITT population with the exclusion of participants who, for reasons other than treatment failure or death, do not complete a protocol-adherent course of treatment. A participant will be considered to have completed a protocol-adherent course of treatment if they have taken 80% of expected doses within 120% of the regimen duration. Participants who receive more than 7 days of either a prohibited concomitant medication or an investigational product not prescribed according to protocol will also be excluded from the PP population.

#### Analysis of the primary endpoint

We will calculate the difference in proportions of participants with a favorable outcome at week 73 between the experimental arm and the control. A one-sided 97.5% confidence interval of the difference will be estimated. The non-inferiority of the experimental arm compared to the control will be established if the difference is greater than the lower equivalence margin, i.e., if the lower bound of the one-sided 97.5% CI is greater than or equal to − 12%. The main primary efficacy analyses will be performed on both mITT and PP populations for the non-inferiority comparison. All of the comparisons performed to demonstrate non-inferiority will be done at the full one-sided alpha level of 2.5%. Adjusted analyses on the primary endpoint will be also performed by controlling for covariates including country, presence of comorbidities, degree of resistance, prior exposure to TB treatment, extent-of-TB-disease phenotype, and BMI. Analyses stratified by country and extent-of-TB-disease phenotype will also be performed. Kaplan–Meier estimates of probability of favorable outcomes will also be generated for the mITT population. Any imbalance between the arms (possibly due to implementation of the amended protocol) will be assessed. No interim analyses nor stopping guidelines are planned. A full description of the statistical methods, including handling of missing data and planned sensitivity analyses, will be detailed in the Statistical Analysis Plan.

### Ethics and dissemination of trial findings

Ethics approval for the study protocol and informed consent materials was granted before the study start from the following entities: MSF Ethics Review Board, Harvard Medical School Institutional Review Board (IRB), IRD IRB, ITM IRB, University of California San Francisco IRB and IRBs/Ethics Committees in all countries in which the study is implemented. Any amendments to the protocol or consent materials are reviewed and approved by all central IRBs, local authorities, and local IRBs before implementation. The trial is registered on ClinicalTrials.gov (NCT03896685). The results of the trial will be disseminated in peer-reviewed publications and at scientific conferences under the responsibility of the Principal Investigators of the study. Investigators/study authors will have full access to the final trial dataset. Trial results will be published in peer-reviewed scientific journals and presented at national and international conferences, as appropriate. Results will be shared and discussed with study participants and affected communities. Authorship will be defined according to International Committee of Medical Journal Editors criteria. No professional writers will be involved. Trial data will be made available to researchers after publication of the primary results through a data sharing platform. The full study protocol and statistical code are available from the corresponding author on reasonable request.

### Future use of biological specimens

Subjects (and their legal representatives as applicable) are asked to provide written informed consent for storage and future use of health information, sputum samples, and *M. tuberculosis* strains isolated from samples. A subject may consent to study participation without consenting to future use of stored specimens, strains, and/or health information. Specimens and strains may be stored in a specimen or strain repository or bank for up to 20 years. Stored specimens, strains, and health information may be used only to improve diagnosis (including resistance testing) and treatment of TB. The specific conditions governing strain and specimen banking are detailed in agreements that comply with intellectual property standards of the Sponsor. All necessary permissions will be obtained before exporting any specimen or strain out of the participating countries according to national regulations in the countries concerned. Relevant IRBs will oversee any future research.

## Discussion

endTB-Q is unique among trials to date for it is a combination of attributes: it is powered to establish non-inferiority in the target population of pre-XDR TB; it is randomized; it uses an internal, concurrent standard-of-care control; and, it assigns duration of treatment according to baseline and on-treatment characteristics. This combination of attributes has important implications for conclusions on safety and efficacy.

The design features implemented in endTB-Q afford the opportunity to evaluate efficacy of an all-oral shortened regimen against the contemporaneous standard of care for pre-XDR TB. This is distinct from previous trials. Nix-TB examined a shortened all-oral regimen of bedaquiline, pretomanid, and linezolid (BPaL) in a population that included patients with fluoroquinolone-resistant pre-XDR TB. It was, however, evaluated against an external, historical control treated with regimens that did not conform to the contemporaneous standard of care for that population, i.e., the comparator regimens excluded bedaquiline, delamanid, pretomanid, and linezolid, and could include fluoroquinolones. Moreover, the higher MICs for pretomanid in lineage 1 strains of *M. tuberculosis* [31], and their higher prevalence in settings with high burdens of pre-XDR TB (i.e., South and Southeast Asia) [32] means that the three-drug regimen could be substantially compromised when linezolid is interrupted for toxicity. BEAT-India examined an all-oral shortened regimen—the same used in the endTB-Q trial—exclusively for pre-XDR TB without an internal, contemporaneous comparator [33]. TB-PRACTECAL was a randomized, internally controlled study of a population including fluoroquinolone-resistant and fluoroquinolone-sensitive MDR/RR-TB. Overall, only 32 participants with pre-XDR TB received the newly recommended BPaLM regimen [34]. All three studies reported encouraging results, with favorable outcomes reported in more than 80%. None, however, could conclusively establish the non-inferiority of an all-oral, shortened regimen compared to the contemporaneous standard of care for fluoroquinolone-resistant MDR/RR-TB. Observational reports reveal that the current standard, a longer regimen containing two Group A drugs and two–three Group B drugs, is outperforming historical treatments for this population [35]. Results from randomized studies are therefore critical to establish whether the endTB-Q shortened regimen strategy has at least comparable efficacy to the current, improved longer standard of care in persons with pre-XDR TB.

Likewise, endTB-Q will address open questions about the toxicity of all-oral, shortened regimens containing newer and repurposed drugs relative to the current standard of care. endTB-Q will contribute to two key toxicity questions. First, endTB-Q will advance knowledge about linezolid dose and duration optimization. Linezolid’s well-established activity coupled with its dose- and duration-limiting toxicity has motivated reduction of cumulative linezolid exposure. Recent studies have tested a range of doses: from the lowest of 600 mg daily for 9 weeks in ZeNix to the highest of 1200 mg daily for 26 weeks in Nix-TB and ZeNix. BEAT-India, TB-PRACTECAL, and ZeNix all contained intermediate doses or durations. Some inter-study differences in reported toxicity align with dosing/duration. But when dosing was similar, differences also occurred. For example, in Nix-TB, 81% of participants experienced peripheral neuropathy, while in ZeNix, only 38% of participants receiving the 1200 mg daily dose for 26 weeks experienced this event. In BEAT-India, linezolid was dosed at 600 mg/day for the full duration of treatment and peripheral neuropathy was reported in 42% of participants; in the comparably dosed group in ZeNix, the event occurred in 24%. These could be the result of different inclusion criteria, severity scales, clinical management, or reporting practices. Only when these are all consistent, or differences can be accounted for in analysis, can valid inference be drawn. The absence of internally controlled studies of sufficient sample size to answer important linezolid-dose optimization questions is reflected in the most recent WHO recommendations on linezolid. Drawing from the ZeNix study, WHO recommends 6 months of 600 mg daily dose of linezolid in the BPaL and BPaLM regimens. This recommendation, however, derives from very small sample sizes with power adequate to detect only very large differences in safety or efficacy among the doses and durations tested. It also deviates from the strategy used in the TB-PRACTECAL, in which linezolid was started at 600 mg daily and reduced to 300 mg daily at 16 weeks. The randomized assignment of linezolid dose-reduction strategies in endTB-Q and endTB clinical trials will provide the required consistency in procedures and larger sample sizes to revisit current recommendations. Second, endTB-Q contributes to evidence on the safety of three QTc interval prolonging drugs—bedaquiline, delamanid, and clofazimine. The experimental regimen contains these drugs and the standard of care is likely to as well. The comparison of this combination in longer vs shorter regimens is novel for safety and efficacy. Emerging evidence from small, controlled trials and uncontrolled studies has supported the concomitant use of bedaquiline and delamanid. In a sample of 84 participants receiving 6 months of treatment, DELIBERATE revealed importantly that QTc interval prolongation with their combined use was no more than additive over that occurring when bedaquiline or delamanid was used without the other. Clofazimine, however, was excluded from participant regimens in DELIBERATE. In BEAT-India, clofazimine was included in the 6–9-month regimen: overall, there were no serious adverse events of QTc prolongation or cardiotoxicity although 27 episodes of grade 3 QTc prolongation were reported among 152 participants. In the endTB observational study, in which 84.5% of participants received bedaquiline, delamanid, and clofazimine as part of longer (18–24 months) conventional regimens, the grade 3 or higher QTc prolongation was less common than other important adverse events and cardiotoxicity was rare. endTB-Q will be the first study to compare concomitant use of these three QTc interval prolonging drugs between short and long MDR/RR-TB regimens.

Evidence on these toxicity-related questions is particularly important in light of the impact that toxicity has had on the relative frequency of unfavorable outcomes in recent studies of regimens for MDR/RR-TB. NExT compared a shortened all-oral regimen for fluoroquinolone-sensitive MDR/RR-TB to the longer standard of care for that same form of disease. Favorable outcomes at treatment completion were 25.0% in the control arm (mITT) and 57.1% in the all-oral, shorter experimental arm (relative risk [RR] 1.9 [95% CI 1.3, 2.7]). Drug substitution due to toxicity was common and was considered to be an unfavorable outcome in the primary analysis. When drug substitution was not used to establish unfavorable outcome, the efficacy benefit of the experimental arm was eliminated (RR 1.0 [95% CI 0.8, 1.3]). In TB-PRACTECAL, the effect of toxicity on unfavorable outcomes is also apparent. In the mITT population of fluoroquinolone-resistant and -sensitive participants, 17/66 (25.8%) participants in the control arm had unfavorable outcomes (early treatment discontinuation) because of an adverse event; in the BPaLM arm, this proportion was 5/55 (9.1%). Since current guidance for pre-XDR TB retains the long, conventional regimen as one of two recommended treatment options, it will be critical to understand the relative contribution of toxicity to outcomes in the endTB-Q experimental strategy compared to the current standard of care.

endTB-Q will also contribute to evidence on duration of shortened regimens for resistant TB. Using a stratified medicine approach informed by recent meta-analyses [36], it prospectively assigns the duration of treatment according to baseline characteristics and treatment response. Accumulating evidence reveals that baseline characteristics (semi-quantitative smear or Xpert grade, lung cavitation on chest radiograph) modify the effect of shortened regimens, when compared to longer regimens for drug-susceptible and for drug-resistant TB. Conventional “one-size-fits-all” treatment durations are often longer than necessary for the majority of patients in order to be of adequate duration for a minority. For that reason, in patients with signs of extensive disease, the endTB-Q experimental regimen is longer than in those without such signs. Initial duration assignment is based on a composite of smear microscopy grade and presence of lung cavitation. Delayed culture conversion has also been identified as a harbinger of risk of treatment failure and relapse; it has been used in STREAM, Nix-TB, and ZeNix to inform treatment prolongation. In endTB-Q, a second evaluation of risk for failure/relapse is performed 6 months after treatment start, taking into account all sputum culture results available at that time. Since failure of treatment (and potential resistance amplification) for these highly resistant forms of TB is an extremely risky prospect for individuals and populations, it is critical to avert these outcomes while awaiting proof-of-principle that duration-randomized designs can estimate optimal duration of TB treatment regimens [37]. Recent WHO guidance reveals some gaps in the evidence available for decision-making around duration of shorter regimens, recommending 6 months for all patients starting the BPaLM regimen but 6 months duration for BPaL, extended to 9 months if sputum cultures are positive at between 4 and 6 months.

Lastly, endTB-Q results are likely to be generalizable across many settings. The included population is heterogeneous, enrolling from 7 epidemiologically distinct countries. Patients with important comorbidities (e.g., diabetes mellitus, viral hepatitis, HIV) are eligible, adolescents may be included, and people who become pregnant during the study can be retained. The inclusion of these subgroups—common among people living with MDR/RR-TB—may permit the use of endTB-Q results to inform clinical guidance for special populations.

Limitations of endTB-Q include its open-label design. Study procedures reduce the impact of this design feature: although participants and Site Investigators are unblinded to treatment assignment, laboratory staff, analysts, and Central Investigators are blinded. The planned comparison to an evolving standard of care is a limitation and an improvement over a design that holds the control static according to outdated recommendations. It complicates interpretation relative to the optimal situation in which all control arm participants received the standard of care in force at trial’s end. However, since phase 3 trials of drug-resistant TB treatment enroll over multiple years and treatment recommendations are now changing nearly annually, it is impossible to guarantee a fully contemporaneous standard of care. Mitigating factors include study procedures that anticipated—and respond to—changes in WHO treatment guidance during trial enrollment and planned subgroup comparisons between experimental arms and different compositions of control arms. The “hybrid” follow-up approach adopted by both the endTB and endTB-Q trials balances the objectives of producing results as rapidly as possible and ensuring the ability to detect most relapse cases in the experimental strategy. This approach is supported by a secondary analysis of time to relapse in TB treatment-shortening trials [38] and it reduces time to study results with modest impact on relapse detection. In the endTB-Q trial, all participants assigned to the experimental strategy will be followed for more than 8 months post-treatment and the vast majority will be followed for more than 12 months.

In conclusion, the endTB-Q trial promises to improve the evidence available for clinical and policy decision-making around the treatment of pre-XDR TB with a four-drug, all-oral, shortened regimen. It builds on the profound advances made in treatment in recent years and strengthens the foundation for future research stratified-medicine designs. By informing recommendations regarding short, effective, safe treatment alternatives for critical, neglected patient subgroups, endTB-Q can contribute to increased access to treatment for this difficult-to-treat form of TB.

## Trial status

endTB-Q recruitment began in the first site in March 2021; with expansion to additional sites, enrolment is expected to be complete in April 2023. The manuscript was submitted prior to the end of recruitment. We submitted it late in the recruitment period because a protocol amendment was pending with several country IRBs and we preferred to submit for publication the final, approved version of the protocol. The protocol was submitted more than 1 year before expected last-patient last-visit. The Journal did not begin the review process until several months after recruitment ended. 

### Supplementary Information

**Additional file 1. **SPIRIT 2013 Checklist: Recommended items to address in a clinical trial protocol and related documents*.

## Data Availability

Not applicable—no identifying images or other personal or clinical details of participants are presented here or will be presented in reports of the trial results.

## Abbreviations

DSMB: Data Safety Monitoring Board
GCP: Good Clinical Practice
HMS: Harvard Medical School
ICH: International Conference on Harmonisation
IRB: Institutional Review Board
IRD: Interactive Research & Development
ITM: Institute of Tropical Medicine
MDR: Multidrug-resistant
mITT: Modified intent-to-treat
MSF: Médecins Sans Frontières
PIH: Partners In Health
PP: Per protocol
RR: Rifampicin-resistant
TB: Tuberculosis
XDR: Extensively drug-resistant
